# EMMPRIN Is an Independent Negative Prognostic Factor for Patients with Astrocytic Glioma

**DOI:** 10.1371/journal.pone.0058069

**Published:** 2013-03-13

**Authors:** Li Tian, Yang Zhang, Yu Chen, Min Cai, Hailong Dong, Lize Xiong

**Affiliations:** 1 Department of Anesthesiology, Xijing Hospital, Fourth Military Medical University, Xi’an, Shaanxi, China; 2 Department of Orthopedic Surgery, Xijing Hospital, Fourth Military Medical University, Xi’an, Shaanxi, China; University of Portsmouth, School of Pharmacy & Biomedical Sciences, United Kingdom

## Abstract

Extracellular matrix metalloproteinase inducer (EMMPRIN), also known as CD147, is a member of the immunoglobulin superfamily that is present on the surface of tumor cells and stimulates adjacent fibroblasts to produce matrix metalloproteinases (MMPs). It has been proved to be associated with tumor invasion and metastasis in various human malignancies. In our study, the protein expression level of EMMPRIN in 306 cases of astrocytic glioma is investigated by immunohistochemistry assay. Statistical analysis was utilized to evaluate the association of EMMPRIN with clinicopathological characteristics and prognosis of patients. It was proved that EMMPRIN protein expression was increased in glioma compared with that in normal brain tissue. Moreover, EMMPRIN immunohistochemical staining was correlated with WHO grade and Karnofsky performance score for strong positive EMMPRIN staining is more frequently detected in glioma of advanced grade or low KPS score. It is also demonstrated that EMMPRIN could be an independent negative prognostic factor in glioma for patients with glioma of strong EMMPRIN staining tend to have high risk of death. These results proved that EMMPRIN is associated with prognosis of glioma, which may also suggest the potential role of EMMPRIN in glioma management.

## Introduction

Glioma is the most common human primary brain tumors with a tendency to invade the surrounding brain tissue, among which astrocytic glioma comprises the largest subgroup [1], [2]. According to World Health Organization (WHO) classification, glioma are histologically classified into four grades: low-grade astrocytomas (WHO grade I–II), anaplastic astrocytomas (WHO grade III), and glioblastoma (GBM, WHO gradeIV) [3], [4]. Even with recent advances in cancer diagnostic methodologies and treatments, prognosis of patients with glioma remains not satisfied [5]. The 5-year survival rate of low-grade glioma is 30% to 70% depending on histology. While glioblastoma, the most aggressive type which usually grows and infiltrates rapidly, has the worst prognosis with median survival time to be 9 to 12 months [6]. Besides the high invasiveness and therapeutic resistance nature, this poor prognosis of glioma could also attributable at least partly to the lack of reliable tumor markers for prognosis and molecular targets against [7], [8]. Thus, identification of prognostic markers might help to assess more precisely the prognosis and to address more clearly the use of adjuvant therapy.

Recent studies have revealed that degradation of the extracellular matrix (ECM) mainly by matrix metalloproteinases (MMPs) is a crucial step for tumor to infiltrate and invade the surrounding normal brain tissue [9], [10]. Extracellular matrix metalloproteinase inducer (EMMPRIN), also known as CD147, is a member of the immunoglobulin family of adhesion molecules and a type I transmembrane glycoprotein [11], [12]. It can stimulate adjacent interstitial normal cells to produce MMPs, which are a group of zinc-dependent proteins known to have the ability to facilitate cell-substrate modulating, tumor invasion and metastasis of epithelial tumor cells by its ECM degrading ability [13], [14]. It is proved that EMMPRIN has an abundant expression in various malignancies including glioma compared with normal tissues [15]. The role of EMMPRIN in tumor invasiveness has also been confirmed immunohistochemically in several types of cancer cells and surrounding tissue [16], [17]. Carcinoma cells can interact with adjacent normal cells to produce MMPs via EMMPRIN on their surface, and, in turn, invade lymphatic tissue and blood vessels and penetrate through the ECM to adjacent organs [18]. Given the important function of EMMPRIN in tumor progression, some reports demonstrated that EMMPRIN correlated with clinical prognosis of various human malignancies such aspulmonary adenocarcinoma, salivary duct carcinoma, prostate cancer, bladder cancer, breast cancer and colorectal cancer [19]–[24]. As far as glioma is concerned, the most common malignancy in human central nerve system, is concerned, the prognostic value of EMMPRIN has only been investigate in pediatric glioma which is different from adult glioma in progression and treatment response due to their different possible molecular mechanism [25], [26]. Therefore, it would be of theoretical and clinical importance to investigate the prognostic role of EMMPRIN in adult glioma to demonstrate its function and clinical utilization potentiality.

In this present study, we have investigated the protein expression of EMMPRIN in clinical glioma specimens, evaluated its association with clinicopathological characteristics and prognosis of patients.

## Materials and Methods

### Patients and Specimens

The present research has been approved by the ethics committee of the Fourth Military Medical University. All patients or family members involved have provided written informed consent. Fresh clinical astrocytic glioma specimens were collected from 306 patients who underwent surgery between January 2004 and December 2006 in Xijing Hospital and Tangdu Hospital, the Fourth Military Medical University. Patients who died of diseases not directly related to glioma had been excluded from this study. None of these patients had received radiotherapy or chemotherapy prior to surgery. Eligible patients with WHO grade I and II glioma received stereotactic fractionated radiotherapy to a total dose of 45 Gy postoperatively; eligible patients with WHO grade III glioma patients received stereotactic fractionated radiotherapy to a total dose of 60 Gy postoperatively; eligible WHO grade IV glioma patients received stereotactic fractionated radiotherapy to a total dose of 60 Gy and three courses of carmustine given at 4-week intervals postoperatively. In addition, normal brain tissue samples were taken from 58 patients who underwent surgery for reasons other than malignancy, such as cerebral trauma; these normal control samples were collected by partial resections of normal brain tissue required as decompression treatment for severe head injury to reduce increased intracranial pressure. The histomorphology of all tissue specimens had been confirmed by the Department of Pathology, the Fourth Military Medical University. Patients’ clinical information, such as age, sex, Karnofsky performance score (KPS) and WHO grade was collected and stored in a database. Follow-up information of all eligible patients was updated every three month by telephone visit and questionnaire letters. Overall survival was calculated from the date of the initial surgical operation to death. Death of participants was ascertained by reporting from the family and verified by review of public records.

### Immunohistochemistry Assay

Immunohistochemistry was performed by avidin-biotin-peroxidase method on all the 306 glioma specimens and 58 normal control specimens. All sections were deparaffinized in xylene and dehydrated through a graduated alcohol series before endogenous peroxidase activity was blocked with 0.5% H_2_O_2_ in methanol for 10 min. Without washing, sections were incubated with mouse monoclonal antibody EMMPRIN (Santa Cruz sc-21746, 1∶200) in PBS at 4°C overnight in a moist box. Negative controls were performed by replacing the primary antibody with pre-immune serum. Biotinylated secondary antibody (1∶400, Sigma) was incubated with the sections for 1 h at room temperature and detected with a streptavidin-peroxidase complex. The brown color indicative of peroxidase activity was obtained by incubating with 0.1% 3, 3-diaminobenzidine (Sigma) in PBS with 0.05% H_2_O_2_ for 5 min at room temperature. Images were obtained under a light microscope (Olympus BX51, Olympus, Japan) equipped with a DP70 digital camera.

### Evaluation of Staining

The EMMPRIN staining was viewed separately by two pathologists without knowing the clinical or clinicopathological status of the cases. The expression of EMMPRIN on slide was evaluated by scanning the entire tissue specimen under low-power magnification (×40), and then confirmed under high-power magnification (×200). An immunoreactivity score (IRS) system was applied. The extensional standard: (1) number of positive stained cell ≤5% scored 0; 6% ∼25% scored 1; 26% ∼50% scored 2; 51%∼75% scored 3 ; >75% scored 4, (2) intensity of stain: colorless scored 0; pallide-flavens scored 1; yellow scored 2; brown scored 3. Multiply (1) and (2). The staining score was stratified as - (0 score, absent), + (1∼4 score, weak), ++ (5∼8 score, moderate) and +++ (9∼12 score, strong) according to the proportion and intensity of positively stained cancer cells. Specimens will be rescored if difference of scores from two pathologists was more than 3.

### Statistical Analysis

Associations between EMMPRIN expression and clinicopathological characteristics were analyzed by Mann-Whitney test and Kruskal-Wallis test, as appropriate. Survival curves were estimated using the Kaplan-Meier method and differences in survival distributions were evaluated by the log-rank test. Cox’s proportional hazards modeling of factors potentially related to survival was performed in order to identify which factors might have a significant influence on survival, and controlling for age, gender and differentiation status. Differences with a *P* value of 0.05 or less were considered to be statistically significant.

## Results

### Immunohistochemical Detection of EMMPRIN Staining

**Figure 1:**
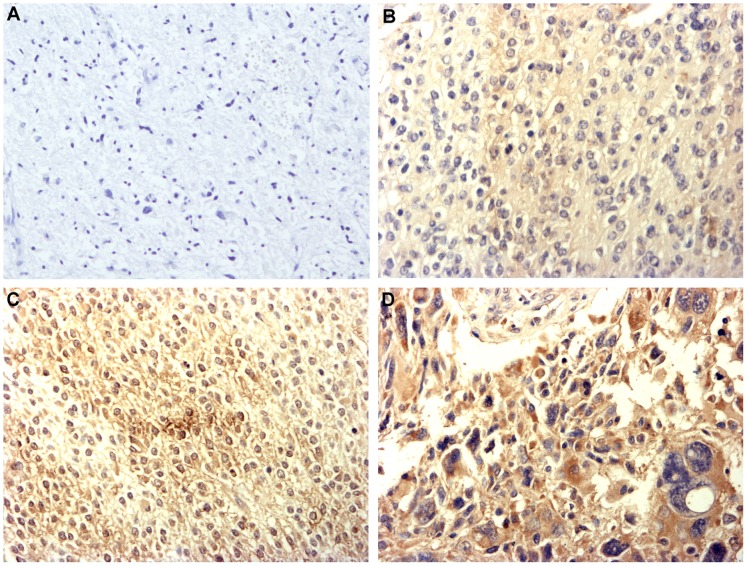
Immunohistochemistry staining pattern of EMMPRIN in glioma (×200). A Negative staining (−) of EMMPRIN, figure was taken from grade III glioma; **B** Weak positive staining (+) of EMMPRIN, figure was taken from grade II glioma; **C** Moderate positive staining (++) of EMMPRIN, figure was taken from grade II glioma; **D** Strong positive staining (+++) of EMMPRIN, figure was taken from grade IV glioma.

In the immunohistochemistry assay, 306 cases of glioma and 58 cases of normal control were investigated. EMMPRIN staining mainly located at cell membrane whereas tumor cell also showed a cytoplasmic EMMPRIN staining, which is in consistence with previous report (Figure 1). Among all the 306 cases of glioma specimens investigated, 49 cases strong positive staining (+++) of EMMPRIN were detected while 75 cases moderate positive staining (++), 106 cases weak positive staining (+) and 76 cases negative staining (−) of EMMPRIN were detected respectively. Whereas among 58 normal control specimens, no strong positive staining (+++) of EMMPRIN was detected while 1 cases moderate positive staining (++), 12 cases weak positive staining (+) and 45 cases negative staining (−) of EMMPRIN were detected respectively. The staining of EMMPRIN in glioma was significantly stronger than in normal control tissues is (*P*<0.001). These results suggested that the expression of EMMPRIN in glioma was increased compared with that in normal control tissues.

### The Relationship of EMMPRIN to Clinicopathological Characteristics

Based on staining evaluation and the statistical analysis, we further analyzed the association of EMMPRIN staining with clinicopathological characteristics of glioma patients. In glioma of different WHO grade, positive ration of EMMPRIN staining tend to increase from grade I to grade IV glioma, suggesting that EMMPRIN staining was significantly associated with WHO grade of glioma (*P<*0.001, Table 1). As far as subgroups were considered, statistical analysis showed that the differences of EMMPRIN staining between subgroups were all significant (*P<*0.05) except that between grade I and grade II glioma (*P* = 0.118). Furthermore, it is also found that the positive ration of EMMPRIN was statistically higher in patients with KPS<80 in comparison to patients with KPS≥80 (*P<*0.001, Table 1). However, the positive ration of EMMPRIN was not found to be associated with patient sex (*P = *0.833) or age (*P = *0.865, Table 1).

**Table 1:** Association of EMMPRIN with clinicopathological characteristics.

	n	EMMPRIN staining				*P*
		–	+	++	+++	
**Total**	306	76	106	75	49	
**Gender**						0.820*
Male	169	40	65	36	28	
Female	137	36	41	39	21	
**Age**						0.865*
≤40	126	32	44	33	17	
40–65	112	27	38	27	20	
≥65	68	17	24	15	12	
**KPS**						<0.001*
≥80	101	39	33	20	9	
<80	205	37	73	55	40	
**WHO grade**						<0.001†
I	40	24	11	3	2	
II	66	30	21	10	5	
III	85	14	35	22	14	
IV	115	8	39	40	28	

* *P* value was estimated by Mann Whitney test.

† *P* value was estimated by Kruskal Wallis test.

### The Relationship of EMMPRIN to Overall Survival

**Figure 2:**
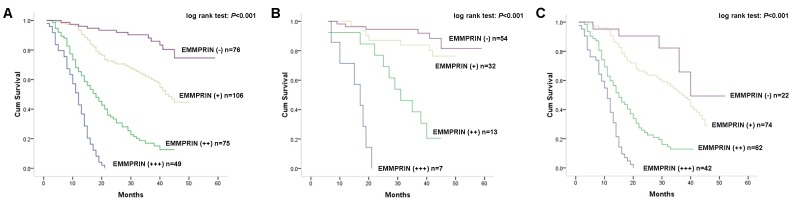
Kaplan-Meier postoperative survival curve and log rank test for patterns of patients with astrocytic glioma and EMMPRIN staining. A Kaplan-Meier analysis and log rank test on all patients (n = 306) proved patients with stronger staining of EMMPRIN had worse prognosis (log rank test: *P*<0 .001); B Kaplan-Meier analysis and log rank test on patients with low grade glioma (n = 106) proved patients with stronger staining of EMMPRIN had worse prognosis (log rank test: *P*<0 .001); C Kaplan-Meier analysis and log rank test on patients with high grade glioma (n = 200) proved patients with stronger staining of EMMPRIN had worse prognosis (log rank test: *P*<0 .001).

During the follow-up period, 178 of the 306 patients (58.2%) with glioma had died. Kaplan-Meier postoperative analysis was used to analyze the survival rate of patients with glioma of hierarchical EMMPRIN staining. Results showed that patients with glioma of stronger EMMPRIN staining tend to have poorer overall survival (log rank test: *P*<0 .001, Figure 2A). The median survival time of patients with negative (−) staining of EMMPRIN could not be estimated for all patients survived longer than overall median survival time. While the postoperative median survival time of patients with weak positive (+) expression of EMMPRIN was 42 months (95% CI: 37.5–46.5), and those of patients with moderate positive (++) and strong positive (+++) of EMMPRIN were 18 months (95% CI: 13.8–22.2) and 12 months (95% CI: 10.1–13.9), respectively. When unadjusted hazard ratio (HR) was considered with EMMPRIN negative (−) staining as reference, patients with glioma of strong positive EMMPRIN staining had a 6.38-fold higher risk of death (95% CI: 3.55–11.48; *P*<0 .001) compared with those with glioma of negative EMMPRIN staining, and the unadjusted HR of moderately positive (++) and weak positive (+) groups were 4.27 (95% CI: 2.75–10.06; *P*<0 .001), and 2.51 (95% CI: 1.39–4.50; *P* = 0 .002). As glioma can be classified into low grade (grade I and II) and high grade (grade III and IV) subtypes according to morphological feature. We further analyzed the prognostic value of EMMPRIN in these two subtypes separately. Results showed that stronger EMMPRIN staining was significantly associated with worse overall survival of low grade glioma patients (log rank test: *P*<0 .001, Figure 2B). Among 106 cases of low grade glioma, the median survival time of patients with negative (−) and weak positive (+) staining of EMMPRIN could not be estimated for all these patients survived better than overall median survival time. While the postoperative median survival time of patients with moderate positive (++) and strong positive (+++) EMMPRIN staining were 31 months (95% CI: 21.6–40.4) and 17 months (95% CI: 11.9–22.1), respectively. While 200 cases of high grade glioma was concerned, it is proved that EMMPRIN was a negative prognostic factor (log rank test: *P*<0 .001, Figure 2C) with median survival time of negative (−), weak positive (+), moderate positive (++) and strong positive (+++) group to be 40 months (95% CI can not be estimated due to limited event number), 37 months (95% CI: 31.2–42.8), 15 months (95% CI: 11.1–18.9) and 11 months (95% CI: 8.9–13.1) respectively.

As far as clinicopathological characteristics were considered, WHO grade was proved to be associated with overall survival since patients with high grade glioma tend to have poorer overall survival and higher risk of death compared with those of low grade (*P*<0 .001). Moreover, age and KPS score were also proved to be associated with survival of patients since patients with older age (age 40–65 *P* = 0.023; age ≥65 *P* = 0.005) or KPS score<80 (*P* = 0.018) tend to have worse overall survival and higher risk of death. However, sex had no prognostic value on overall survival of patients with glioma (Table 2).

**Table 2:** Association of molecular and clinical factors with prognosis of patients.

	Unadjusted HR* (95% CI)	*P*	Adjusted HR† (95% CI)	*P*
**EMMPRIN**				
Negative (−)	–		–	
Weak positive (+)	2.51 (1.39–4.50)	0.002	2.57 (1.41–4.83)	0.001
Moderate positive (++)	4.27 (2.75–10.06)	<0.001	4.52 (2.88–10.96)	<0.001
Strong positive (+++)	6.38 (3.55–11.48)	<0.001	6.61 (3.62–13.21)	<0.001
**Sex**				
Female	–		–	
Male	1.21 (0.91–2.19)	0.203	1.25 (0.93–2.31)	0.192
**Age**				
≤40	–		–	
40–65	1.72 (1.21–3.29)	0.023	1.86 (1.27–3.55)	0.015
≥65	2.67 (1.43–5.02)	0.005	2.95 (1.92–5.86)	0.002
**KPS score**				
≥80	–		–	
<80	1.81 (1.29–3.68)	0.018	1.76 (1.25–3.37)	0.024
**WHO grade**				
I	–		–	
II	2.55 (1.63–3.84)	<0.001	2.38 (1.57–3.61)	<0.001
III	7.32 (4.21–14.53)	<0.001	7.03 (3.81–13.92)	<0.001
IV	13.21 (7.42–25.63)	<0.001	12.26 (6.11–23.71)	<0.001

* Hazard ratios in univariate models.

† Hazard ratios in multivariable models.

In multivariate analysis, Cox proportional hazards model was adjusted for sex, age, KPS score, WHO grade and treatment. Results showed that the adjusted HR of weak positive (+), moderate positive (++) and strong positive (+++) EMMPRIN groups were 2.57 (95% CI: 1.41–4.83; *P* = 0 .001), 4.52 (95% CI: 2.88–10.96; *P*<0 .001), and 6.61 (95% CI: 3.62–13.21; *P*<0 .001) respectively. These results proved that EMMPRIN was an independent prognostic factor of overall survival for patients with glioma. Thus, increased EMMPRIN expression could be an indicator of poor overall survival without consideration of age, sex, KPS score or WHO grade. In addition, age, KPS score and WHO grade were also proved to be independent prognostic factors for overall survival (Table 2).

## Discussion

Recent studies suggested that a diversity of biological changes in glioma cells may account for the poor overall survival of patients with glioma [27]. And several clinicopathologic features have been considered as important prognostic factors for glioma, such as age at diagnosis, WHO grade and KPS score. However, these factors may not estimate prognosis in glioma patients accurately because of patients’ heterogeneity for the outcome in each grade is highly variable and genetic differences among them may also contribute to their different survival. Thus, regular treatments do not benefit all patients equally and adverse effects of the treatments may also dramatically deteriorate the quality-of-life of some patients. Thus, it is hoped that a greater understanding of molecular factor involved in glioma prognosis will lead to new insights into accurate prognostic prediction, which is critical to the selection of appropriate therapeutic approaches.

The primary purpose of the present study is to determine the prognostic value of EMMPRIN on overall survival of patients with glioma. Results proved that the protein expression of EMMPRIN in glioma was increased compared with that in normal brain, which is consistent with previous investigation [25], [26]. It has also been proved that EMMPRIN mRNA and protein were both significantly higher in glioma than in normal brain, indicating the consistency of EMMPRIN mRNA and protein expression in glioma and normal brain [28]. These results suggested that EMMPRIN might play an oncogenic role in human glioma.

In addition, EMMPRIN expression was statistically associated with WHO grade of glioma for strong EMMPRIN staining was more frequently detected in glioma of advanced grade. Previous study on mRNA level also showed the same trend that EMMPRIN mRNA expression was correlated with tumor progression since it was the highest in grade IV glioma, followed by grade III and low grade glioma [28]. These results indicated increasing trend of EMMPRIN expression as the grade of glioma go up, which might account for partially the elevation of invasion and metastasis ability of glioma with advanced grade. When other clinicopathological characteristics of patients were considered, EMMPRIN expression was also proved to be associated with KPS score of patients for EMMPRIN expression were higher in patients with KPS<80 compared with those with KPS≥80, suggesting that the function of EMMPRIN might affect clinical behavior of patients with glioma. However, EMMPRIN was not found to be associated with patients’ gender or age.

Moreover, we further analyzed the prognostic role of EMMPRIN on overall survival of patients with glioma. Kaplan-Meier analysis showed a significant association between EMMPRIN expression and overall survival of patients that patients with stronger EMMPRIN staining had a shorter survival time. As glioma can be classified into low grade and high grade subtypes according to morphological features. We also analyzed the prognostic value of EMMPRIN in these two subtypes separately. Results showed that stronger EMMPRIN staining was significantly associated with worse overall survival in both low grade and high grade glioma. These findings proved that EMMPRIN could be a negative prognostic factor for patients with glioma irrespective of WHO grade. Clinicopathological including older age, KPS<80 and advanced WHO grade were also proved to be associated with worse overall survival of patients. Cox proportional hazards model adjusted for age, gender, KPS score, WHO grade and treatment showed the same trend as Kaplan-Meier analysis, indicating that EMMPRIN could be an independent prognostic factor for patient with glioma. These results suggested that EMMPRIN could constitute a useful prognostic marker to identify patients who are more likely to have disease recurrence and are, thus, good candidates to receive an aggressive treatment. However, future prospective studies are needed to determine its accuracy and efficiency on predicting the prognosis of patients with glioma in order to tailor treatment.

The mechanism lie behind this association might be diverse. In human glioma, MMPs stimulated by glioma cell EMMPRIN may be one of these mechanisms. MMPs can enhance tumor cell invasion by degrading extracellular matrix proteins, activating signal transduction cascades that promote motility and solubilizing extracellular matrix-bound growth factors in various human malignancies including glioma [9], [10], [26]. Consequently, EMMPRIN may induce tumor invasion and metastasis by activating the production of MMPs through modulating cell–substrate and adhesion processes. It has been demonstrated that silencing CD147 by RNA interference approach could inhibit tumor progression in murine lymphoid neoplasm and pancreatic cancer [29], [30]. Moreover, it is proved that EMMPRIN can regulate malignant cell proliferation, migration, anchorage-independent growth, and cell survival via the activation of ERK1/2 and p38 mitogenactivated protein kinases [31]. It is also indicated that down-regulation of EMMPRIN by RNA interference could result in decreased X-linked inhibitor of apoptosis (XIAP) expression and an anti-tumor effect through enhancing the susceptibility of cancer cells to apoptosis [32]. And a recent study has also shown that EMMPRIN has the ability to enhance tumor angiogenesis via its regulation on the expression of vascular endothelial growth receptor (VEGF) [33]. Moreover, MMPs stimulated by EMMPRIN can even regulate tumor cell behavior through a large variety of other signaling molecules [34].

Our study proved that EMMPRIN expression is related to glioma WHO grade, KPS score and overall survival of patients. EMMPRIN was also proved to be an independent prognostic factor for overall survival of patients with glioma, which supported the notion it may be a molecule involved in tumor invasion and metastasis.
